# Ab Externo Choroidal Fluid Drainage, Pars Plana Vitrectomy, and Endotamponade for the Management of Persistent Hypotony following Glaucoma Surgery

**DOI:** 10.1155/2024/5323632

**Published:** 2024-07-29

**Authors:** Susanna Friederike Koenig, Efstathios Vounotrypidis, Christian Maximilian Wertheimer, Armin Wolf

**Affiliations:** Augenklinik der Universität Ulm, Prittwitzstrasse 43 89075, Ulm, Germany

## Abstract

**Background:**

Persistent severe serous choroidal detachment is a rare complication after glaucoma surgery. Surgical treatment with choroidal fluid drainage through a scleral incision is an option in these cases. Combining this procedure with pars plana vitrectomy and gas endotamponade has potential advantages. In the following, the perioperative course of this surgical option in a small cohort will be presented.

**Methods:**

This is a retrospective cohort study of the postoperative course of ab externo drainage of persistent serous choroidal detachment (≥4 weeks) in combination with pars plana vitrectomy and gas endotamponade in six eyes of six patients after exhausting all conservative treatment options. Inclusion criterion was persistent hypotony with severe serous choroidal detachment after intraocular pressure (IOP) lowering surgery due to medically uncontrolled glaucoma. Eyes were evaluated according to resolution of choroidal detachment, change in IOP and visual acuity (VA), postdrainage complications, and need for further surgeries.

**Results:**

Before surgery, all patients presented with flat anterior chamber, decreased vision, and persistent choroidal detachment. The surgery itself was uneventful, but due to the complexity of the cases, tailoring the procedure to each patient's needs was required. Complete resolution of choroidal effusion was achieved by one month in 5 eyes and in 1 eye by month 3. There was an increase in average IOP from 5 (±2.1) mmHg before surgery to 11.3 (±3.7) mmHg and in VA from 1.7 (±0.8) to 1.2 (±0.6) logMAR. Five out of six patients required additional surgery, mainly to further increase the IOP even though choroidal detachment had already resolved.

**Conclusion:**

Ab externo choroidal fluid drainage combined with pars plana vitrectomy and gas endotamponade seems to be an effective and safe treatment option in persistent ocular hypotony. Although repeated surgeries might be necessary, large-scale prospective studies must be undertaken to provide corroborative evidence.

## 1. Introduction

Clinically significant ocular hypotony (≤5 mmHg and vision loss) after glaucoma filtration surgery is a rare, but potentially sight-threatening condition [1]. It occurs in 7.9–50% after penetrating glaucoma surgery and bears the risk of developing a serous choroidal detachment (CD) [2–4]. It is associated with the use of antifibrotic, glaucoma drainage devices, bleb leakage, and iatrogenic cyclodialysis cleft formation [5, 6]. Findings include hypotony maculopathy, accelerated cataract formation, optic neuropathy, astigmatism, corneal edema, and Descemet membrane folds [7, 8]. Serous choroidal detachments in particular must be mentioned, as they can lead to decreased visual acuity, a flat anterior chamber, and devastating nontraumatic suprachoroidal hemorrhage [9].

However, nonpenetrating glaucoma surgery is associated with a lower risk of postoperative hypotony. There are several preventive measures to reduce the risk of choroidal exudation, such as releasable sutures, laser suture lysis techniques, or the use of viscoelastics. If choroidal effusion does occur, surgical treatment most often is not necessary, especially in the absence of relevant complications, and medical treatment regularly results in resolution of effusion, normalization of intraocular pressure (IOP), and visual acuity (VA) [10, 11].

In cases where conservative management fails and choroidal detachment persists, various surgical options are available in order to restore function and anatomy of the eye. Serous fluid drainage can be drained by pressurizing the anterior chamber by means of viscoelastic [12]. Ab externo drainage with pressurizing the anterior chamber alone has often been described [13–17].

Nevertheless, there is no general surgical approach to long lasting severe hypotony changes in literature, especially after glaucoma surgery. Ab externo drainage of suprachoroidal fluid in combination with pars plana vitrectomy and gas endotamponade has potential advantages, which will be discussed in this paper on the basis of six patients with persistent serous choroidal detachment associated with ocular hypotony after glaucoma surgery, who underwent this procedure.

## 2. Methods

### 2.1. Study Design

Six eyes of six patients were included in this retrospective observational study. All suffered from persistent serous choroidal detachment (≥4 weeks) after glaucoma surgery that resulted in hypotony, as well as a shallow anterior chamber with iridocorneal contact and corneal hypotony changes. Eyes were initially treated with topical cycloplegics, topical or systemic steroids, and anterior chamber (AC) injections of viscoelastic. Patients were identified by retrospective chart review of all glaucoma patients between April 1, 2020 and December 31, 2022. The study site was the Department of Ophthalmology, University Eye Hospital, Ulm, Germany, and the study was approved by the Local Ethics Committee (approval ID: 52/23).

### 2.2. Decision to Operate

After all conservative and less invasive treatment options (injections of viscoelastic into the AC) were exhausted and a mean of 10.7 ± 16.5 months (range: 1–43 months) after the primary surgery, all six eyes still presented with a flat anterior chamber with iridocorneal contact, hypotony keratopathy with corneal edema and Descement membrane folds, serous choroidal effusion with kissing bullae, and a mean IOP of 5 (±2.1) mmHg. In three patients, the IOP was higher than 5 mmHg; however, they presented with hypotonic changes to the better-seeing eye, including vast kissing choroidal detachment. At this time, it was decided to perform an ab externo choroidal drainage combined with pars plana vitrectomy and gas endotamponade.

### 2.3. Surgical Technique

All procedures were performed by a single advanced vitreoretinal surgeon (AW). The shallow anterior chamber was deepened with balanced salt solution through a corneal incision at a bottle height of 50 mmHg, which was kept constant throughout the procedure (Oertli OS4, Oertli Instruments, Switzerland). A conjunctival incision was made inferiorly depending on the location of the maximum elevation of choroidal detachment. After cauterization of episcleral vessels, a lamellar sclerotomy of 2–3 mm was made using a 15° blade approximately 6–10 mm posterior to the limbus. Spontaneous fluid drainage occurred in all cases. To allow further drainage, a spatula was inserted through the incision to hold it open until no more fluid was released. The sclerotomy was left open to allow further intra- and postoperative drainage, the conjunctiva was sutured at a later point during vitrectomy, and a standard 23-gauge three-port vitrectomy was performed with gas fill after fluid-air exchange. At first, a paracentesis was performed temporally at the limbus and an anterior chamber maintainer was placed to ensure controlled IOP during trocar placement. After that, a 23 g trocar was inserted at 3.5 mm posterior to the limbus as flat as possible at the temporal inferior quadrant to avoid any insertion into the CD area. After checking of the trocar being in the vitreous cavity using the light pipe, as well as indentation, the AC maintainer was removed, the infusion cannula was connected to the trocar, and irrigation was set to 40 mmHg. Two more 23 g trocars were inserted in the same way at the superior temporal and nasal quadrant 3.5 mm posterior of the limbus. Carefully, a core pars plana vitrectomy was performed under visualization of the CD. Induction of posterior vitreous detachment was induced centrally around the optic nerve head, if not present. Vitreous was further detached to the equator, depending on the extension of CD. Vitreous base shaving and endolaser if necessary were performed when CD had diminished at a later point of the surgery. Under endoillumination transconjunctival exodrainage using a 27 g needle was additionally performed at the place of the highest CD at an IOP of 45 mmHg. At the end, a fluid-air exchange followed by injection of a gas endotamponade (SF6, 20%) was performed, followed by suturing all scleral incisions with 8-0-vicryl sutures to ensure that all wounds were sealed.

### 2.4. Follow-Up

**Figure 1:**
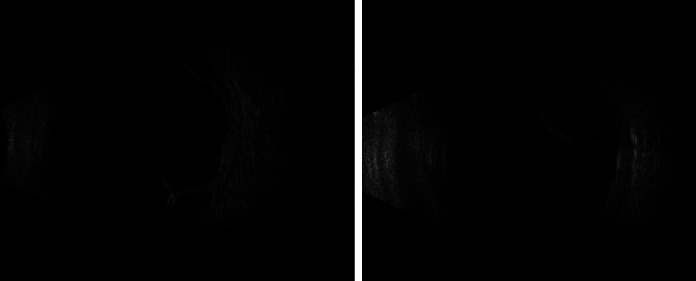
B-scans of different eyes showing persistent serous choroidal detachment.

**Figure 2:**
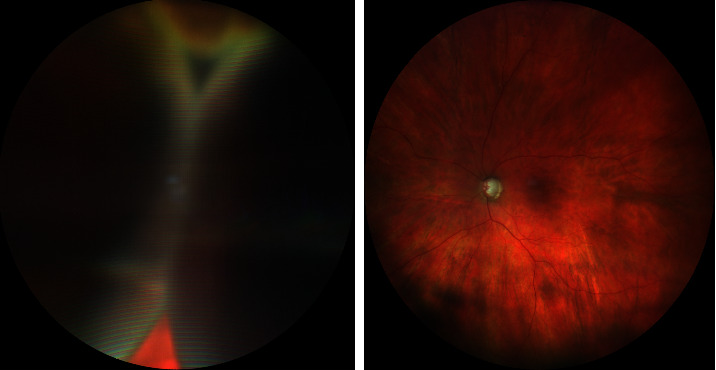
Fundus photographs of persistent serous choroidal detachment in one patient at presentation and after resolution after ab externo drainage combined with vitrectomy and gas endotamponade.

After surgery, patients were asked to remain in supine position for at least four days to allow the anterior choroid to approximate to the sclera assuring a buoyancy induced counterpressure to the ciliary body. All patients had regular outpatient visits to our clinic before and after surgery for at least six months postoperatively, after which their follow-up visits were scheduled with their primary ophthalmologist. Data recorded included ocular and general history, slit-lamp examination of the anterior and posterior segments, type of surgery leading to the choroidal detachment, reason for treatment decision, VA, IOP (assessed by means of Goldmann applanation tonometry), ultrasound B scans of the eye (Figure 1) to document and qualify choroidal detachment prior to surgery as well as postoperatively, fundus photographs (Figure 2), and additional surgical procedures.

### 2.5. Endpoints

Successful outcome was defined as complete resolution of serous choroidal detachment including IOP elevation to a normal level. Secondary outcomes included visual acuity and intra and postoperative complications, including secondary surgeries.

### 2.6. Statistical Analysis

Excel 365 (Microsoft, Redmond, WA, USA) was used for data collection and processing. When a mean was calculated, the standard deviation was reported as the error.

## 3. Results

### 3.1. Patient Characteristics

The mean age was 65.5 (±17.4) years with a range of 38–83 years. Four out of six patients were male, and all were Caucasian. The mean IOP prior to the hypotony causing glaucoma surgery was 32.2 (±6.1) mmHg on an average of 3.2 (±0.4) topical medications and one patient receiving only oral acetazolamide. The glaucoma procedures included Ahmed FP7 valve implantation in four patients and transscleral cyclophotocoagulation in two patients.

### 3.2. Surgery

No patient had suprachoroidal blood identified prior to surgery. In two patients, suprachoroidal blood was detected when performing surgical drainage. Otherwise, surgery was uneventful except for a partial zonulolysis and a small retinal break, which was treated with endolaser. Pars plana vitrectomy peeling of centrally located tractional membranes, endodiathermy, and peripheral retinotomy were performed in one patient with choroidal detachment associated with circular tractional retinal detachment.

### 3.3. Follow-up

A slight increase in IOP was observed immediately after surgery, and complete resolution of the serous choroidal effusion occurred at one month in five patients and at three months in one patient. Hypotony keratopathy was resolved within one to two weeks and the AC without shallowing. Secondary procedures were common and were performed the earliest six weeks after surgery. Only one patient remained without further procedures. An intravitreal dexamethasone implant was injected in one case and intracameral viscoelastic in another to increase IOP. In one eye, a XEN implant was placed in the Ahmed tube to reduce and restrict aqueous flow and increase IOP and one Ahmed tube was ligated with nonabsorbable sutures. One patient required another pars plana vitrectomy for a retinal hole. Six months after surgical drainage of the choroidal effusions, mean IOP was 11.3 (±3.7) mmHg without medication and visual acuity was significantly reduced but often improved (Table 1).

## 4. Discussion

This study presents six eyes of six patients with ab externo drainage of serous suprachoroidal fluid combined with pars plana vitrectomy and gas endotamponade for the management of persistent serous choroidal detachment in ocular hypotony after glaucoma surgery.

This procedure was chosen because vitrectomy with gas tamponade may offer certain potential advantages. These are due to the surface tension of the endotamponade, which exerts a force on both the location of the serous detachment and the ciliary body intra- and postoperatively. Choroidal effusions are thought of as tissue edema and can be explained by Starling's law; hence, relative hydrostatic and oncotic pressures across the choroidal capillary membrane may be increased, resulting in less effusion. A decrease in IOP leads to accumulation of fluid in interstitial spaces. Furthermore, inflammation increases the permeability of choroidal membranes. As a result of this, it is believed that there is increased transudation through the choroidal capillary walls [18].

Hypotony often leads to flattening of the anterior chamber. Without vitreous, the risk for additional flattening of the anterior chamber may also be reduced. Of course, one could have tried an AC pressurization combined with CD drainage; however, there is no sufficient evidence regarding the correct surgical procedure. In order to perform a more controlled surgical procedure and since one surgeon (AW) performed all interventions, it was decided to combine the procedure with pars plana vitrectomy and gas endotamponade (SF6: 20%).

Gas endotamponades are associated with IOP-elevation after vitrectomy in up to 58.9% of the cases [19, 20]. In our study, the reason for choosing a gas endotamponade was to assure that IOP will be at least within normal boundaries or at the upper normal levels to allow quicker CD resolution, as long as the gas remains in the eye. Furthermore, all incisions were sutured to avoid gas migration or loss and make sure that no hypotony will occur at the early postoperative period [21].

With this technique, we were able to treat the persistent choroidal effusion in all our patients.

Surgical drainage of persistent suprachoroidal fluid has been described previously. These studies used sclerostomy alone without pars plana vitrectomy and reported successful results [15, 22, 23]. A study of 63 eyes of 63 patients who underwent one or more choroidal drainage procedures after glaucoma surgery showed complete resolution with a low complication rate. Cataracts were shown to progress after choroidal drainage, which was also discussed as being due to hypotony prior to drainage and could have an impact on BCVA improvement after surgery [15]. However, in our study, only one patient was phakic (patient 5). In another study of 112 eyes that underwent surgical drainage of ciliochoroidal detachment after glaucoma surgery, many eyes required repeated surgeries as well [16]. The evidence for outcome from these older studies is limited due to the rarity of the disease, and further studies are needed to determine the optimal approach, especially in light of more modern vitrectomy techniques.

In literature, pars plana vitrectomy has been used to treat persistent hypotony in cases unrelated to glaucoma surgery. Although the conditions treated are different from those in our study, they share at least a part of the pathophysiology. In eyes with combined rhegmatogenous retinal detachment and choroidal detachment, pars plana vitrectomy alone without choroidal drainage was successful [24]. In idiopathic uveal effusion syndrome, pars plana vitrectomy was used to treat choroidal effusion [25]. In uveitic hypotony, pars plana vitrectomy with removal of ciliary membranes alone was sufficient to restore IOP in some cases [26]. Cyclodialysis, which is common after ocular trauma, has been described to be successfully treated by pars plana vitrectomy and gas tamponade [27]. In these cases, the anterior gas tamponade can aid postoperative reattachment of the ciliary body and correct aqueous hyposecretion [28]. The role of pars plana vitrectomy in glaucoma surgery and hypotony remains unclear, but there is evidence to support further studies to evaluate its role.

Although an attempt has been made to standardize the surgical procedure described, diagnosis, medical history, and other factors vary widely among our patients, and an individualized approach must be taken to each patient's needs in these challenging cases. For example, the following situations have arisen and have been managed accordingly. Pars plana vitrectomy peeling of centrally located tractional membranes, endodiathermy, and peripheral retinotomy were performed in one patient with choroidal detachment associated with circular tractional retinal detachment. Another patient underwent previous transscleral cyclophotocoagulation for secondary glaucoma due to Sturge–Weber syndrome, which always carries a high risk of developing postoperative hypotony [29]. Particularly fragile hemangiomatous vessels in the choroid have a tendency to exudate in these eyes after IOP reduction [30]. In four of our six patients, overfiltration through an Ahmed valve was the precipitating factor. After choroidal drainage surgery, two patients required additional procedures to raise the IOP. One was managed by placing a XEN implant inside the tube of an Ahmed valve to reduce the tube diameter and thus the flow, which has also been described in pediatric glaucoma management [31]. The other Ahmed tube was ligated externally with a permanent suture. Interestingly, two patients had a hemorrhagic component that could not be detected preoperatively by ultrasound or examination.

Limitations of our study are the lack of a noninterventional group and the retrospective approach. Follow-up is limited, mainly due to the policy of referral to a local ophthalmologist from our university-based tertiary center some time after the surgery. A small case number is presented due to the rarity of the disease and the fact that almost all patients with ocular hypotony after glaucoma surgery can be successfully managed with medical treatment to increase IOP and visual acuity and lead to resolution of choroidal effusions [32]. Furthermore, this study focused on the surgical procedure and the postoperative period, so risk factors for the development of persistent ocular hypotony could not be determined.

## 5. Conclusion

In conclusion, vitrectomy and drainage of serous choroidal detachment seem to be an effective and safe treatment option in persistent ocular hypotony and persistent serous choroidal detachment. Although repeated surgeries might be necessary to restore visual function, IOP, and the anatomy of the eye itself, large-scale prospective studies must be undertaken to provide corroborative evidence.

## Figures and Tables

**Table 1:** Summary of patient characteristics.

Patient	Age/sex	IOP prior to glaucoma surgery (mmHg)	Number of IOP-lowering medications	Diagnosis	Type of glaucoma surgery	VA preop (logMAR)	VA postop (logMAR) after 6 months	Time from glaucoma surgery to drainage surgery (months)	IOP predrainage (mmHg)	IOP postdrainage (mmHg) after 6 months
1	m/82	39	3	POAG, tractional retinal detachment	Cyclodiode laser	4.0 (LP)	3.0 (HM)	43	2	7
2	m/69	39	Oral acetazolamide only	Uveitic glaucoma, serous retinal detachment, choroidal detachment	Ahmed FP7	3.0 (HM)	0.7	13	5	8
3	f/60	32	4	POAG	Ahmed FP7	2.3 (CF)	0.7	2	7	13
4	m/79	28	3	Pseudoexfoliation glaucoma	Ahmed FP7	0.7	1.3	2	6	10
5	m/38	32	3	Secondary glaucoma in naevus flammaeus	Cyclodiode laser	1.0	1.1	1	7	17
6	f/83	23.4	3	POAG	Ahmed FP7	1.4	1.3	3	3	13

POAG: primary open-angle glaucoma, VA: visual acuity, HM: hand movement, LP: light perception, CF: counting fingers.

## Data Availability

All data generated or analysed during this study are included within the article.
